# IDENTIFYING NEW INTERVENTION TARGETS TO REDUCE RISK OF ELDER ABUSE AND NEGLECT IN DEMENTIA FAMILY CAREGIVING

**DOI:** 10.1093/geroni/igad104.2016

**Published:** 2023-12-21

**Authors:** Carolyn Pickering, Vicki Winstead, Melba Hernandez-Tejada

## Abstract

Persons living with dementia experience higher rates of abuse and neglect than their cognitively intact peers. Caregiver burden, including stress related to management of behavioral symptoms, has been repeatedly shown as a risk factor for family caregivers’ use of abusive and neglectful behavior. However, interventions intended to reduce caregiver burden and stress and improve management of behavioral symptoms often produce only small effect sizes and are not successful in translation. Therefore, it is important to identify other modifiable risk factors to serve as intervention targets to reduce and prevent elder abuse and neglect in dementia family caregivers. The purpose of this symposium is to understand the role of three potential targets for which there are efficacious interventions available: alcohol consumption, family conflict and self-compassion. The first paper presents the differential impact of screening positive for alcohol use disorder and daily drinking patterns on risk of neglect, physical and psychological abuse. The second paper examines the role of family conflict on a caregiver’s use of abusive and neglectful behaviors, including how risk varies by the relationship type (e.g., adult child caregiver vs spouse). The third paper discusses the protective effect of self-compassion on daily risk of abuse and neglect, including the buffering effect it has on daily stress appraisal. The discussant will highlight implications and future directions for intervention research, including how the field may shift to a personalized approach to intervention delivery to match the right intervention to the right person.

